# Biennial surveillance of *Plasmodium falciparum* anti-malarial drug resistance markers in Democratic Republic of Congo, 2017 and 2019

**DOI:** 10.1186/s12879-022-07112-z

**Published:** 2022-02-10

**Authors:** Doudou M. Yobi, Nadine K. Kayiba, Dieudonné M. Mvumbi, Raphael Boreux, Pius Z. Kabututu, Pierre Z. Akilimali, Hippolyte N. T. Situakibanza, Patrick De Mol, Niko Speybroeck, Georges L. Mvumbi, Marie-Pierre Hayette

**Affiliations:** 1 Department of Basic Sciences, Faculty of Medicine, University of Kinshasa, Kinshasa, Democratic Republic of Congo; 2 School of Public Health & Research Institute of Health and Society, Catholic University of Louvain, 1200 Brussels, Belgium; 3 School of Public Health, Faculty of Medicine, University of Kinshasa, Kinshasa, Democratic Republic of Congo; 4 Department of Public Health, Faculty of Medicine, University of Mbujimayi, Mbuji-Mayi, Democratic Republic of Congo; 5 Laboratory of Clinical Microbiology, University of Liège, 4000 Liège, Belgium; 6 Department of Internal Medicine, Faculty of Medicine, University of Kinshasa, Kinshasa, Democratic Republic of Congo

**Keywords:** Biennial, Surveillance, *Plasmodium falciparum*, Anti-malarial, Resistance, Markers, Democratic Republic of Congo

## Abstract

**Background:**

Because of the loss of chloroquine (CQ) effectiveness, the Democratic Republic of Congo (DRC)’s malaria treatment policy replaced CQ by sulfadoxine–pyrimethamine (SP) as first-line treatment of uncomplicated malaria in 2003, which in turn was replaced by artemisinin-based combination therapies (ACT) in 2005. The World Health Organization (WHO) recommends monitoring of anti-malarial drug resistance every 2 years. The study aimed to provide baseline data for biennial molecular surveillance of anti-malarial drug resistance by comparing data from a study conducted in 2019 to previously published data from a similar study conducted in 2017 in the DRC.

**Methods:**

From July to November 2019, a cross-sectional study was conducted in ten sites which were previously selected for a similar study conducted in 2017 across the DRC. *P. falciparum* malaria was diagnosed by a rapid diagnostic test (RDT) or by microscopy and dried blood samples (DBS) were taken from patients who had a positive test. Segments of interest in *pfcrt* and *pfk13* genes were amplified by conventional PCR before sequencing.

**Results:**

Out of 1087 enrolled patients, 906 (83.3%) were PCR-confirmed for *P. falciparum*. Like in the 2017-study, none of the mutations known to be associated with Artemisinine (ART) resistance in Southeast Asia was detected. However, non-synonymous (NS) mutations with unknown functions were observed among which, A578S was detected in both 2017 and 2019-studies. The overall prevalence of *pfcrt*-K76T mutation that confers CQ-resistance was 22.7% in 2019-study compared to 28.5% in 2017-study (p-value = 0.069), but there was high variability between sites in the two studies. Like in 2017-study, the *pfcrt* 72–76 SVMNT haplotype associated with resistance to amodiaquine was not detected.

**Conclusion:**

The study reported, within 2 years, the non-presence of molecular markers currently known to be associated with resistance to ART and to AQ in *P. falciparum* isolated in the DRC. However, the presence of polymorphisms with as-yet unknown functions was observed, requiring further characterization. Moreover, an overall decrease in the prevalence of CQ-resistance marker was observed in the DRC, but this prevalence remained highly variable from region to region.

**Supplementary Information:**

The online version contains supplementary material available at 10.1186/s12879-022-07112-z.

## Background

The spread of resistance to anti-malarial drugs poses a serious public health risk and a real challenge for malaria control in endemic countries. As result of high treatment failure rate (> 10%) with chloroquine (CQ) and sulfadoxine–pyrimethamine (SP) and following the World Health Organization (WHO) recommendation, the Democratic republic of Congo (DRC) malaria national policy implemented artemisinin-based Combination Therapies (ACT) as first-line treatment of uncomplicated *Plasmodium falciparum* malaria in 2005 [1, 2]. WHO recommends biennial surveillance of anti-malarial drug efficacy to ensure early detection of emergence and spread of resistance. Therapeutic efficacy studies (TESs) are the gold standard for the monitoring of anti-malarial efficacy. However, today, in addition to TETs, the assessment of molecular marker in *P. falciparum* genes associated with anti-malarial drug resistance provides useful information about the emergence and spread of resistant parasites [3].

Point mutations in the chloroquine resistance transporter gene (*pfcrt*), notably mutations in amino acids position 72 to 76 have been found to confer resistance to both CQ and amodiaquine (AQ) [4]. The amino acid substitution from lysine (K) to threonine (T) at position 76 of the PFCRT protein (K76T) has been shown to confer CQ resistance [5], while the 72–76 PFCRT haplotype, namely SVMNT (Serine–Valine–Methionine–Asparagine–Threonine) has been linked to AQ resistance [6]. K76T mutation, which is considered as the most reliable molecular marker of CQ resistance, is found in all CQ-resistant isolates of *P. falciparum* [5]. The decline in this CQ-resistance marker following the official CQ removal from national treatment guidelines varies significantly between countries. In several countries, it was reported that after the discontinuance of CQ use, K76T-mutant parasites have been replaced by wild ones, resulting in the decrease in prevalence of K76T marker [7–10]. In other countries, studies reported the persistence of resistant strains several years after stopping the use of CQ as first-line treatment of uncomplicated malaria [11, 12]. In the DRC, the prevalence of K76T marker was 55.4% in a study conducted with samples obtained from 2007 Demographic and Health Survey (2007-DHS) [13] and 63.9% in a study conducted in 2014 [14]. Moreover, a large variability in this prevalence of K76T was observed across the DRC in 2017, with presence of very high prevalence (89.5%) in one province and very low (1.5%) in another [15].

Single-nucleotide polymorphisms (SNPs) occurring within *P. falciparum Kelch* 13 gene (*pfk13*), precisely in the propeller domain region, have been described as molecular markers of ART resistance [16, 17]. The list of these SNPs categorized as validated mutations and candidate mutations of ART resistance is updated by the WHO [18]. Originally, these mutations have emerged and spread in Southeast Asia, but nowadays, some of them are increasingly detected in sub-Saharan African countries providing evidence for de novo emergence of resistance to ART in Sub-Saharan African countries [19–22].

To help improve long-term monitoring of anti-malarial drug resistance, the present study aimed to provide baseline data for biennial molecular surveillance of *P. falciparum* anti-malarial drug resistance by comparing data from the study conducted in 2019 to previously published data from a similar study conducted in 2017 in the DRC [15, 23].

## Methods

### Study population and blood samples collection

**Fig. 1:**
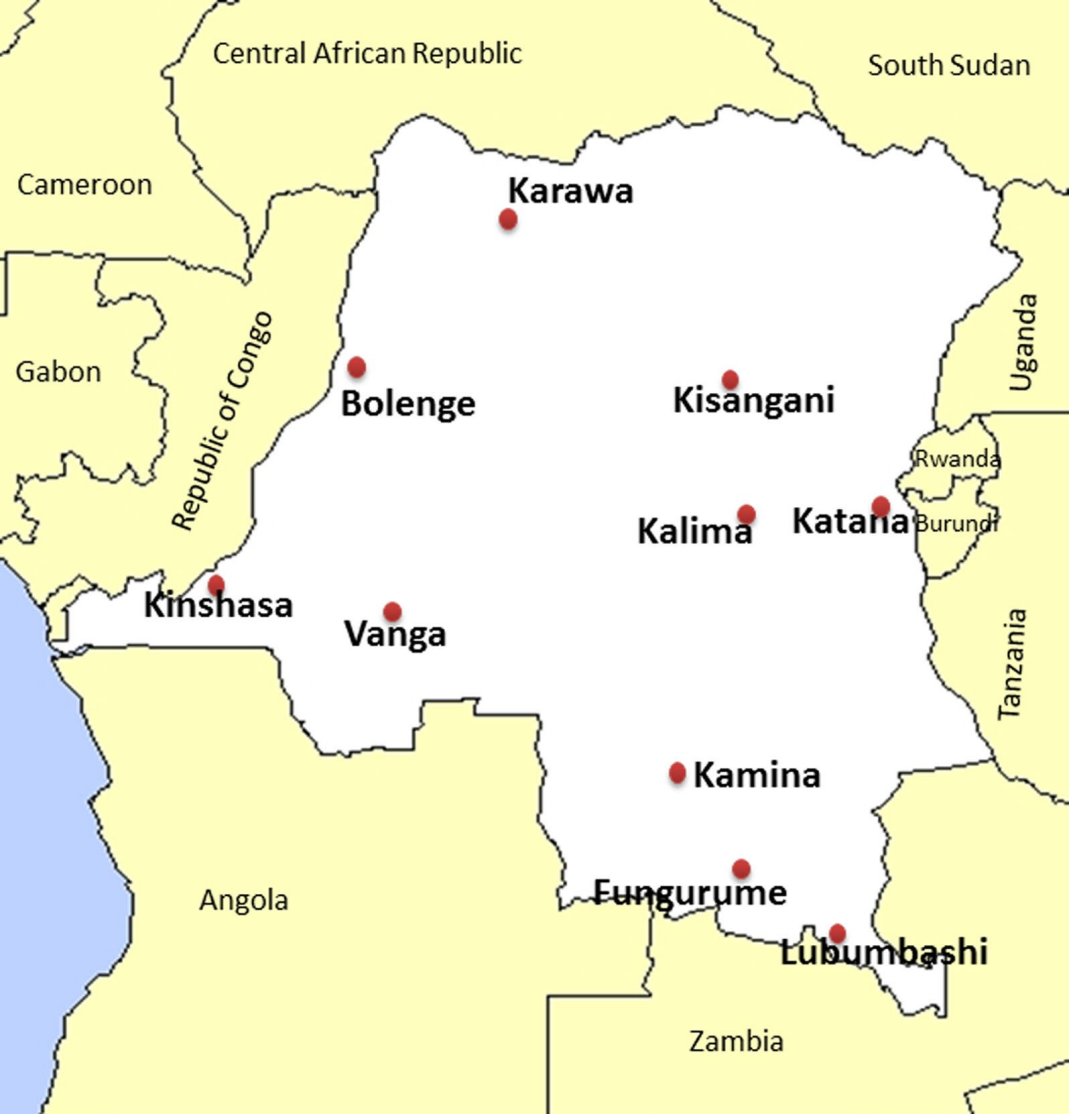
Geographical distribution of study sites across the Democratic republic of Congo (outline map: http://johan.lemarchand.free.fr/cartes/cartes-republique-democratique-du-congo.html)

From July to November 2019, a cross-sectional study was conducted in ten sites (sentinel sites of National Malaria Control Programme) located in ten provinces: Kingasani in Kinshasa, Kabondo in Kisangani/Tshopo, Lubumbashi in Haut-Katanga, Bolenge in Equateur, Karawa in Nord-Ubangi, Vanga in Kwilu, Kalima in Maniema, Kamina in Lomami, Fungurume in Lwalaba and Katana in Sud-Kivu (Fig. 1). *P. falciparum* malaria was diagnosed using a rapid diagnostic test (RDT) or the microscopy in all patients with fever or history of fever in the last 24 h, who presented at one of the selected health structures of each study site. The RDT used was the one available on the site during sample collection (SD Bioline Malaria Ag *P.f*/Standard Diagnostics/South Korea or CareStart Malaria *Pf*/Access Bio/South Korea) while the light microscopy of Giemsa-stained thick blood test was used in some referral health structures where it was feasible. To participate in the study, informed consent was obtained from each adult patient (≥ 18 years old) or from a parent or legal guardian for children and adolescents. Blood samples were collected on filter paper as dried blood spots (DBS) and then transported at the University of Kinshasa for storage at – 20 °C until use.

### Molecular analysis

Extraction of genomic DNA from DBS was carried out using the QIAamp DNA Mini Kit (QIAGEN GmbH, Germany) according to the manufacturer’s instruction (https://www.qiagen.com). Briefly, the DNA purification was performed following the manufacturer’s manual procedure which included four steps: cell lysis, DNA binding to the QIAamp membrane, washing contaminants and DNA elution. The extracted DNA was kept at − 20 °C until time of use. To detect *P. falciparum*, a real-time Pf-PCR assay was performed according to a previously described procedure [24], which was previously modified [15]. Samples with PCR-confirmed presence of *P. falciparum* were used to amplify segments of interest on *pfk13* and *pfcrt* genes. The procedures previously described were used to amplify the interest segment on *pfcrt* gene [25] and that on the propeller region of *pfk13* gene [23]. The analysis of *P. falciparum* genes polymorphisms was done by DNA bi-directional sequencing using Sanger method as previously described [15, 23]. Nucleotides sequences were analyzed using Vector NTI (Thermo Fisher Scientific, US) and aligned to the reference annotated PF3D7 (PF3D7_0709000 for PFCRT sequences and PF3D7_1343700 for PFK13 sequences) to assess SNPs.

### Data analysis

Data were encoded and verified in a Microsoft Excel 2010 database and imported in SPSS Statistics 20.0 for analysis. Only isolates successfully sequenced have been considered to calculate the prevalence of each SNP. Frequencies were statistically compared using Chi-squared tests. Secondary analyses were conducted using logistic regression model to determine if the marginal associations from the univariate model matched those of the multivariate model. The p-values less than 0.05 were considered significant.

## Results

### Baseline characteristics of enrolled patients from 2019-study compared to 2017-study

In total, 1087 patients were enrolled in 2019-study in comparison to 1070 in 2017-study. Their age ranged from 0 to 77 years old. Table 1 presents the distribution of patients according to different characteristics in comparison to 2017-study patients. The distribution per age range showed a low difference (p-value = 0.01) between 2017-study and 2019-study while no difference was observed when considered per sex. Among enrolled patients, 906 (83.3%) were confirmed positive for *P. falciparum* by real-time PCR in 2019-study versus 805 (75.3%) in 2017-study (p < 0.001).

**Table 1:** Characteristics of enrolled patients from 2019-study compared to 2017-study

Characteristic	2017-study		2019-study		p-value
	N	%	N	%	
Age (year)					0.010
0–5	465	43.5	437	40.2	
6–12	259	24.2	225	20.7	
13–19	107	10.0	127	11.7	
20 and +	239	22.3	298	27.4	
Total	1070		1087		
Sex					0.649
Female	551	51.5	573	52.5	
Male	519	48.5	519	47.5	
Site					0.138
Bolenge	98	9.2	99	8.7	
Fungurume	92	8.6	100	8.8	
Kalima	97	9.1	100	8.8	
Kamina	98	9.2	99	8.7	
Karawa	97	9.1	104	9.2	
Katana	105	9.8	87	7.7	
Kingasani	160	15.0	150	13.2	
Kisangani	128	12.0	135	11.9	
Lubumbashi	101	9.4	157	13.8	
Vanga	94	8.8	104	9.2	
Number episode of malaria last 12 months					< 0.001
0	529	49.4	458	40.4	
1	119	11.1	295	26.0	
2	221	20.7	170	15.0	
3	122	11.4	123	10.8	
4 and +	79	7.4	89	7.8	
*P. falciparum* PCR					< 0.001
Negative	264	24.7	181	16.7	
Positive	805	75.3	906	83.3	
Total	1069	100.0	1087	100.0	

N: Number

### Prevalence of chloroquine resistance marker according to patients’ characteristics from 2017-study and 2019-study

As shown in Table 2, the prevalence of CQ-resistance marker was not different between age groups and sex categories (p > 0.05). Globally, this prevalence decreased from 28.5% in 2017-study to 22.7% in 2019-study (p = 0.007), but it was highly variable between sites.

**Table 2:** Chloroquine resistance marker and patient characteristics from merged 2017/2019 studies

	CQ-resistance		No CQ-resistance		p-value
	N	%	N	%	
Age (year)					0.163
0–5	153	23.2	506	76.8	
6–12	108	29.3	260	70.7	
13–19	48	27.9	124	72.1	
20 and +	93	26.1	264	73.9	
Total	402	25.8	1154	74.2	
Sex					0.689
Female	208	25.5	608	74.5	
Male	196	26.4	547	73.6	
Total	404	25.9	1155	74.1	
Site					< 0.001
Bolenge	35	26.5	97	73.5	
Fungurume	2	1.5	132	98.5	
Kalima	7	4.8	140	95.2	
Kamina	14	9.2	138	90.8	
Karawa	17	12.4	120	87.6	
Katana	132	86.8	20	13.2	
Kingasani	112	42.4	152	57.6	
KIsangani	10	5.6	170	94.4	
Lubumbashi	6	4.4	131	95.6	
Vanga	72	44.7	89	55.3	
Number episode of malaria last 12 months					0.001
1	54	18.6	236	81.4	
2	71	26.5	197	73.5	
3	51	28.3	129	71.7	
4 and +	52	38.2	84	61.8	
Total	407	25.5	1189	74.5	
Study					0.007
2017-study	218	28.5	545	71.4	
2019-study	189	22.7	644	77.3	
Total	407	25.5	1189	74.5	

CQ: chloroquine, N: number

### *Pfk13*-propeller polymorphisms and PfCRT-K76T mutation per site in comparison of 2019-study to 2017-study

**Fig. 2:**
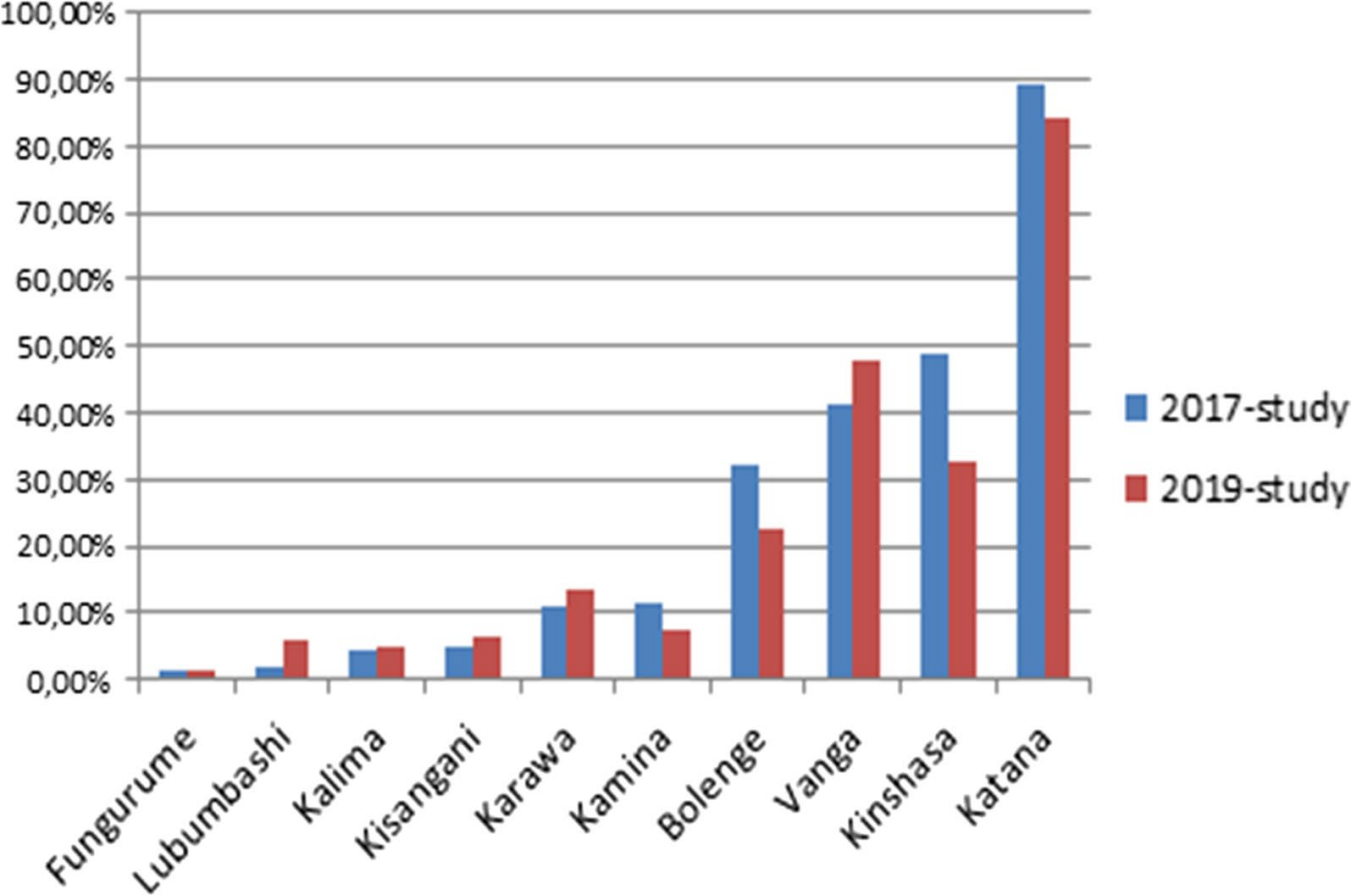
Distribution of K76T mutation from 2019-study versus 2017-study site per site across Democratic Republic of Congo showing the persistence of high variability of the CQ-resistance marker prevalence between sites

In 2019-study, among 822 isolates successfully sequenced on *pfk13*-propeller gene, following non-synonymous mutations have been detected: M472L, D516A, V568M, S576T, A578S, D584E and Y588C (Additional file 1: Table S1), A578S and D584E were each found in two isolates. A578S mutation was the only one detected in both 2017 and 2019-studies in Fungurume and in Kinshasa while all other mutations were detected once (p < 0.001). On *pfcrt* gene, when considered in the same site, the prevalence of K76T mutation did not shown significant difference between 2019-study and 2017-study (p > 0.05), except in Kinshasa (Table 3), while when comparing site by site, this prevalence remained highly variable in the two studies ranging from 1.5 to 89.5% in 2017-study and from 1.4 to 84.2% in 2019-study respectively in Fungurume and in Katana (p < 0.001) (Fig. 2). The 72–76 PfCRT CVIET haplotype was the most prevalent among the K76T-mutant parasites (93.6% in 2019-study versus 95.9% in 2017-study), while the SVMNT haplotype was detected neither by the 2019-study nor the 2017-study.

**Table 3:** PfCRT-K76T mutation and *pfk13*-propeller polymorphisms per site and per study

Site	*pfcrt*-K72T			*pfk13*mutation	
	2017-study n/N (%)	2019-study n/N (%)	p-value	2017-study	2019-study
Bolenge	18/56 (32.1)	17/76 (22.4)	0.21	A578S	
Fungurume	1/65 (1.5)	1/69 (1.4)	0.99	A578S	A578S
Kalima	3/66 (4.5)	4/81 (4.9)	0.99	–	
Kamina	8/71 (11.3)	6/81 (7.4)	0.42	A569T	D584E, V568M
Karawa	6/54 (11.1)	11/83 (13.3)	0.71	–	D584E
Katana	68/76 (89.5)	64/76 (84.2)	0.33	M472I	S576T
Kinshasa	78/160 (48.8)	34/104 (32.7)	0.01	A578S	A578S, M472L
Kisangani	4/85 (4.7)	6/95 (6.3)	0.89	A578S	D516A
Lubumbashi	1/56 (1.8)	5/82 (6.1)	0.43	–	Y588C
Vanga	31/75 (41.3)	41/86 (47.7)	0.42	V534A	
Total	218/764 (28.5)	189/833 (22.7)	0.007		

Pf: *Plasmodium falciparum,* n: Number of isolates carrying K76T, N: Number of isolates successfully sequenced

### Chloroquine resistance marker and patients’ characteristics by logistic regression model

Unlike univariate model, the multivariate logistic regression model showed no statistical difference between the prevalence of CQ-resistance from 2017-study and that from 2019-study (p = 0.069; OR = 0.76; IC 95%: 0.56–1.02). However, the between sites variability of this prevalence has been confirmed by the logistic regression model, as shown in Table 4.

**Table 4:** Chloroquine resistance marker and patients’ characteristics by logistic regression

	Chloroquine resistance		p-value
	OR	IC95%	
Age (year)			
0–5	1		
6–12	1.04	0.72–1.48	0.825
13–19	0.94	0.57–1.55	0.812
20 and +	0.89	0.60–1.33	0.601
Sex			
Female	1		
Male	1.08	0.81–1.45	0.584
Site			
Bolenge	1		
Fungurume	0.04	0.01–0.18	0.000
Kalima	0.13	0.05–0.31	0.000
Kamina	0.19	0.09–0.42	0.000
Karawa	0.33	0.17–0.65	0.001
Katana	17.52	9.36–32.78	0.000
Kingasani	1.90	1.18–3.06	0.008
Kisangani	0.14	0.06–0.31	0.000
Lubumbashi	0.12	0.05–0.32	0.000
Vanga	2.33	1.40–3.89	0.001
Number episode of malaria last 12 months			
0	1		
1	1.03	0.65–1.63	0.886
2	1.15	0.77–1.71	0.469
3	0.11	0.64–1.59	0.961
4 and +	1.64	0.98–2.75	0.057
Study			
Study-2017	1		
Study-2019	0.76	0.56–1.021.02	0.069

## Discussion

Like in 2017-study [23], the 2019-study did not detect any polymorphisms that are currently known to be associated with resistance to ART. However, other coding substitutions observed, such as A578S, should be considered. A578S mutation, commonly reported in African countries including the DRC [23, 26, 27], was the only one which was detected in both 2019-study and 2017-study. Although, the amino acid substitution in A578S mutation is likely to alter the function of PFK13 protein [28], previous studies have not shown the link with ART resistance [17, 27]. The function of the *pfk13* gene is largely unknown and the phenotypic expression of this gene could involve other determinants which could vary from one geographical region to another due to the genetic diversity of *P falciparum*. Other unusual polymorphisms have been observed by both the 2019-study and 2017-study; some of them (M472I, V534A and A569T) were previously reported in African countries [26]. Since the polymorphisms associated with ART resistance are numerous and occur on a background of genetic diversity, the mutations that are increasingly observed in Africa merit further characterization [29, 30].

Globally, the prevalence of the K76T mutation known to be associated with CQ-resistance continues to decline in the DRC. Indeed, compared to previously reported prevalence which was more than 50% [13, 14, 25], the present study has shown that the prevalence of CQ-resistance marker has decreased to less than 30% (28.5% in 2017-study and 22.7% in 2019-study) in the DRC. However, this prevalence remained very variable from one region to another, with the highest prevalence in Katana (89.5% in 2017-study and 84.2% in 2019-study) and the lowest in Fungurume (1.5% in 2017-study and 1.4% in 2019-study). A possible illicit use of CQ that contributes in maintaining CQ selective pressure on the local *P. falciparum* population could explain the persistence of high CQ resistance rate in some regions. In addition, the selection for CQ-resistance is largely dependent on the genetic structure of parasite populations, which have been shown to vary greatly between regions of the DRC [30]; this may partly explain the observed regional variability in prevalence of CQ-resistance rate. Other potential explanations for the heterogeneity in prevalence of CQ-resistance marker in different regions of the DRC have been addressed in the previous 2017-study [15].

The return of CQ-susceptible malaria may present a novel opportunity for reintroducing this molecule to prevent malaria, especially in vulnerable populations. Indeed, if the proportion of CQ-resistant parasites declines in African endemic countries to an undetectable level, the reintroduction of CQ to be used alone or in combination with other anti-malarial drugs for prophylaxis or treatment may possibly be considered. The absence of drug pressure is supposed to be the key driver in the reemergence of CQ-sensitivity parasites in the field setting [31]. Unfortunately, with the advent of the COVID-19 pandemic, CQ has been largely used for its supposed antiviral properties in affected countries including the DRC [32]. In this context, CQ has been made widely available in private drugstores with the presence of falsified version containing low amount of CQ, which was discovered in the DRC [33]. This is likely to maintain CQ pressure on *P*. *falciparum* populations, thus compromising the hope towards the return of CQ-susceptible malaria in endemic countries particularly in the DRC.

The PFCRT 72–76 haplotype, SVMNT has been correlated with high-level resistance to the AQ metabolite desethylamodiaquine (DEAQ) in in vitro tests [34]. Like in 2017-study, the SVMNT haplotype associated with AQ-resistance was not detected in 2019-study. AQ is one of the partner drugs of ACTs used in many endemic countries like the DRC for the treatment of uncomplicated *P. falciparum* malaria. Generally, AQ was found to be effective in Africa, even in the face of increasing CQ-resistance [35] that would constitute a risk factor of emergence of AQ-resistance in Africa. However, high prevalence of SVMNT has been reported in *P. falciparum* from South America and Asia [36] and the presence of this haplotype was also reported in Tanzania [37] and in Angola [38]. AQ is structurally related to CQ with both belonging to the group of 4-aminoquinolines. There is a possibility that AQ could continue to contribute to selection for the mutant *pfcrt-K76T* parasites even after discontinuance of CQ usage [39].

The present study contributed to ongoing malaria surveillance providing baseline data for the biennial surveillance of *P. falciparum* molecular markers associated with resistance to anti-malarial drugs in the DRC. Regular studies with size of samples taking into account the regional variability and a broader exploration of the genome of *P. falciparum* could help to provide more information about the landscape of *P. falciparum* drug resistance in the DRC.

## Conclusions

The study did not report, within 2 years, the presence of molecular markers currently known to be associated with resistance to ART and to AQ in *P. falciparum* isolated in the DRC. Moreover, the overall prevalence of CQ-resistance marker continues to decrease in the DRC, but there was observed a high variability from region to region with persistence of high prevalence of parasites carrying CQ-resistant haplotype. Further characterization of unusual detected polymorphisms is required and regular surveillance should continue in order to ensure early detection and containment of the emerging anti-malarial drug resistance in the DRC.

## Supplementary Information

**Additional file 1**: **Table S1.** *Plasmodium falciparum* K13 nucleotide sequences from 2019-study.

## Data Availability

The datasets used and/or analysed during the current study are available from the corresponding author on reasonable request.

## Abbreviations

ACT: Artemisinin-based combination therapy
AL: Artemether–lumefantrine
AQ: Amodiaquine
ASAQ: Artesunate–amodiaquine
CQ: Chloroquine
DBS: Dried blood spot
DNA: Deoxyribonucleic acid
DRC: Democratic Republic of Congo
NMCP: National Malaria Control Programme
PCR: Polymerase chain reaction
*pfcrt*: *Plasmodium falciparum* Chloroquine resistance transporter
*pfk13*: *Plasmodium falciparum* Kelch 13
US: United States of America
RDT: Rapid diagnostic test
SNP: Single nucleotide polymorphisms
SP: Sulfadoxine–pyrimethamine
WHO: World Health Organization
